# Photoluminescence measurements of carbon quantum dots within three-dimensional hydrogel matrices using a high throughput 96 well plate method

**DOI:** 10.1016/j.mex.2019.02.014

**Published:** 2019-02-26

**Authors:** Adam Truskewycz, Sabrina Beker, Andrew S. Ball, Ivan Cole

**Affiliations:** a School of Science, RMIT University, Melbourne, VIC, 3000, Australia; b Advanced Manufacturing and Fabrication, School of Engineering, RMIT University, Melbourne, VIC, 3000, Australia

**Keywords:** High throughput sensing of heavy metals using fluorescent hydrogels, Hydrogel, High throughput, QCD, Sensing, Hexavalent chromium

## Abstract

Solid or liquid platforms have been traditionally employed for measuring the fluorescent properties of quantum carbon dots (QCD). Hydrogels possess both liquid and solid properties which allow them to overcome several shortfalls of both solid and liquid sensing platforms. Hydrogels offer a three dimensional platform which can house nanoparticles with different attributes (i.e. fluorescent QCD’s) and prevents their aggregation. Here, we incorporate QCD’s (made from the hydrothermal treatment of 1-naphthylamine and citric acid) into the matrix of a zinc oxide hydrogel. This nanocomposite was shown to have hexavalent chromium (Cr^6+^) specific fluorescence quenching properties. Detailed fluorescence analysis of the hydrogel with Cr^6+^ was conducted in a high throughput manner by loading the hydrogel into wells of a black 96-well plate. Fluorescence quenching of the hydrogel-QCD-nanocomposites in the presence of dilutions of Cr^6+^ was measured using a fluorescence spectrophotometer and showed incremental fluorescence decreases with increasing Cr^6+^ concentration. Furthermore, this was quantitatively confirmed by Stern–Volmer plots showing a linear quenching trend (R^2^ = 0.9975) when comparing fluorescence intensities against increasing Cr^6+^ concentrations (0.234–1.875 μM). This technology can be applied for routine water quality testing in agricultural, natural and potable water sources for the early detection of heavy metal pollutants.

## Graphical abstract

**Figure:**
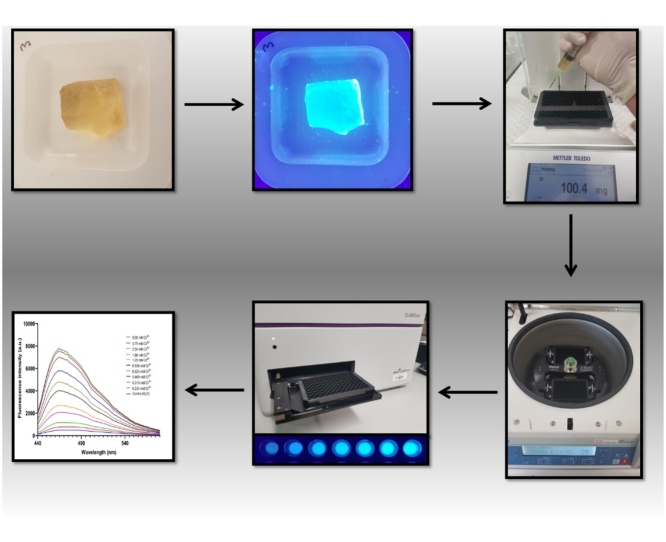

**Specifications Table**

**Table:** 

**Subject area:**	Materials Science
**More specific subject area:**	Environmental sensing of heavy metals
**Method name:**	High throughput sensing of heavy metals using fluorescent hydrogels
**Name and reference of original method:**	NA
**Resource availability:**	NA

## Method details

### Method summary

1 Synthesis of fluorescent quantum dots and transparent hydrogel.
2 Blending of QCD’s and hydrogel to create hydrogel-QCD-composite.
3 Adding of hydrogel-QCD-composite to wells of black 96-well plate for high throughput fluorescence measurements.
4 Addition of sample of interest (i.e. Cr^6+^) which will interact with hydrogel-QCD-composite.
5 Quantitative measurements of fluorescence quenching in the presence of differing Cr^6+^ concentrations.
6 Verification of this sensing platforms validity.

### Quantum carbon dot (QCD) synthesis

In a typical synthesis;

1 A carbon source i.e. citric acid (5.4 g) and an amine/amino source i.e. 1-naphthylamine (1.0 g) are dissolved in ultrapure water (18 MΩ).
2 The solution is transferred to a Teflon-lined autoclave and heated to 200 °C for 2.5 h with a ramp up of 6 °C/min (the temperature and time of heating can be altered to produce QCD’s with different sizes and photoluminescence profiles).
3 The autoclave is allowed to cool to room temperature within a fume hood and the solution is centrifuged at 5000 × *g* for 10 min.
4 The resulting supernatant is filtered through a 0.22 μm syringe filter and put into a dialysis membrane (MWCO 500 Da) for 48 h with ultrapure water (18 MΩ) as the purification solution. This water is changed every 8 h.
5 Following purification, the solution is frozen to −80 °C and subjected to lyophilisation until a dry powder is obtained.

**Note:** hydrothermal synthesis of QCD’s using carbon and amine precursors have been previously generated by a number of different studies [[1], [2], [3]].

### Hydrogel synthesis

In a typical synthesis;

1 Zinc chloride (2.0 g) is dissolved in pure methanol (14.0 mL) and polyvinylpyrrolidone (360,000 MW) (1.0 g) is added and allowed to equilibrate with the zinc electrolyte solution for 1 h.
2 This solution is then placed into a Teflon lined autoclave and heated for 2 h at 200 °C.
3 The autoclave is cooled to room temperature within a fume hood and the pellet is placed into ultrapure water (18 MΩ) so that the unreacted precursors can diffuse out.
4 The water is replaced every 12 h for 3 days to ensure the swollen hydrogel is free of unreacted salts.

### Adding QCD’s to the hydrogel and loading of 96 well plates

In a typical synthesis;

1 Serial dilution of QCD’s in ultrapure water (18 MΩ) are conducted (starting concentration is 20 mg/mL).
2 An aliquot (150 μL) of these dilutions are transferred into black 96 well plates and maximum excitation and emissions profiles recorded using a CLARIOstar® (BMG LABTECH) fluorescence spectrophotometer.
3 The concentration of quantum dots which produces the highest photoluminescence output at its maximum fluorescence excitation wavelength is recorded.
4 Quantum dots are added to ultrapure water (18 MΩ) in the same concentrations that were determined for maximum photoluminescence output.
5 Purified swollen hydrogel is soaked in quantum dot solution and allowed to equilibrate for 24 h.
6 The hydrogel is then rinsed in ultrapure water (18 MΩ) and pat-dried with tissue paper.
7 The solid hydrogel is put into a sterile plastic syringe and a syringe needle is attached to the end. The hydrogel is pushed through the syringe needle twice to ensure that a homogenous hydrogel puree is achieved.
8 To black 96 well plates, 100 mg of homogenous hydrogel puree is loaded into each well using the syringe. A deviation in weight of ±5% should not be exceeded.
9 This 96 well plate containing QCD-hydrogel is then put into a centrifuge capable of holding 96 well plates and is centrifuged at 2000 × *g* to ensure even topography of hydrogel in each well.

**Note:** Fluorescent nanomaterials have been previously incorporated into hydrogels by a number of different studies [[4], [5], [6]]

### Fluorescence measurements

1 Using a fluorescence spectrophotometer equipped with a 96 well plate reader, select ‘scan over emissions’ and set the fluorescence excitation wavelength to the lowest measurable value (in the CLARIOstar®, BMG LABTECH this is 320 nm).
2 Scan the sample using this excitation wavelength (320 nm) allowing the emissions range to extend from 340 nm to 740 nm.
3 Repeat the previous steps but increase the excitation wavelength by 10 nm in each run (i.e. excitation of 330, 340, 350, 360, 370 nm etc.). Note: the emissions range will need to be adjusted from 20 nm over each new selected excitation wavelength to 740 nm.
4 The maximum photoluminescence intensity found over the different excitation wavelengths represents the maximum excitation wavelength and is used for all further fluorescence measurements.
5 Hydrogels by themselves or hydrogels with a water-soluble solution of interacting analytes can be measured by exciting them with their maximum excitation wavelength and recording their photoluminescent emissions between ‘excitation wavelength +20 nm–740 nm.
6 Each measurement should be run with a minimum of three replicates for statistical analysis purposes.

### Method validation

**Fig. 1:**
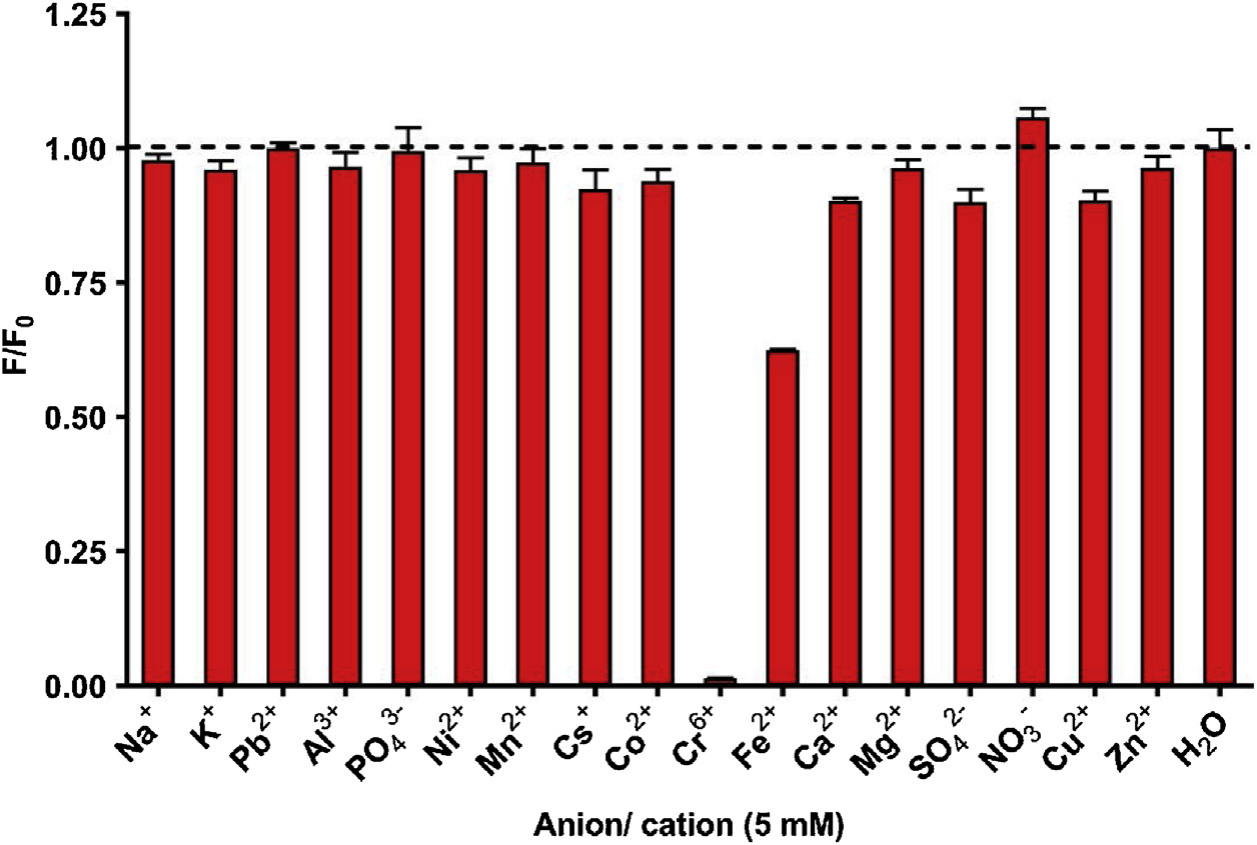
Metal ion selectivity of the hydrogel-QCD-nanocomposite based on their photoluminescence profiles in the presence of different anions/cations (5 mM).

To determine the capacity for hydrogel-QCD-nanocomposites to be used as an accurate, high throughput and functional sensing platform, various fluorescence quenching measurements were performed. Firstly, the hydrogel-QCD-nanocomposite was tested for its capacity to selectively detect hexavalent chromium ions. In triplicate, 5 mM of different anions/cations were added to wells in a 96 welled plate containing 100 mg of hydrogel-QCD-nanocomposite. As can be seen from Fig. 1, the relative fluorescence of most anions/cations is comparable to that of the un-amended water sample. Hexavalent chromium and iron(II) were exceptions to this trend with significantly different photoluminescence outputs. Hexavalent chromium showed significant fluorescence quenching and was considerably lower than all other samples. Low variations in photoluminescence outputs within replicates reinforce the repeatability and accuracy of the measurements.

**Fig. 2:**
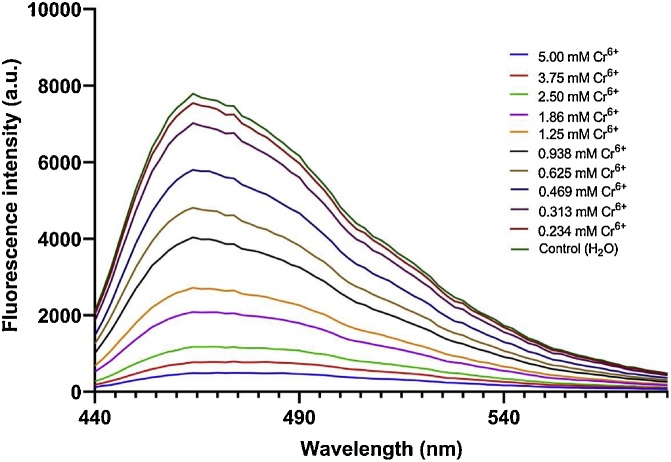
Emission spectra of the Hydrogel-QCD-composite in the presence of various concentrations of Cr6+ in river water.

**Fig. 3:**
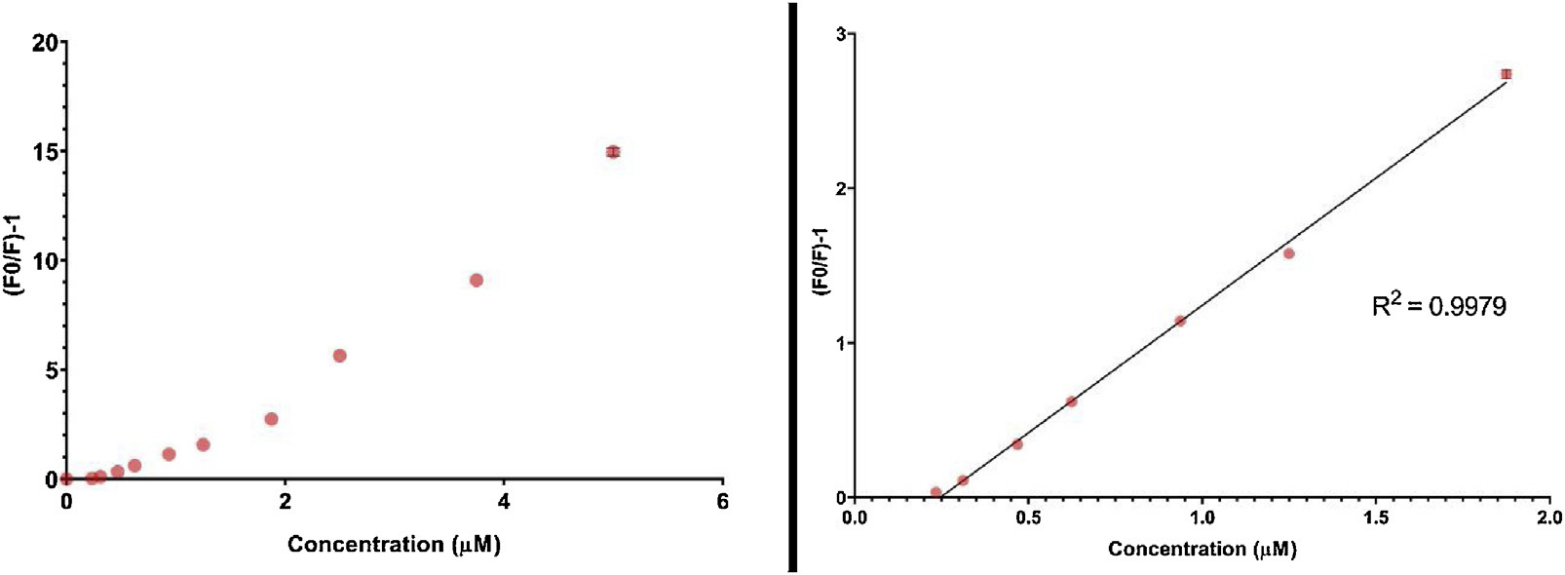
(A) Stern–Volmer plots for the (F/F0)-1 values Cr^6+^ concentrations 0.0–5.0 mM) (B) Stern–Volmer plots for the (F/F0)-1 values Cr^6+^ concentrations 0.234–1.875 μM).

Upon showing that hexavalent chromium could interact with the hydrogel-QCD-nanocomposite and reduce its photoluminescence output, the capacity for the composite to detect low concentrations of hexavalent chromium was assessed. Serial dilutions of hexavalent chromium were created and introduced to the hydrogel-QCD-nanocomposite-loaded 96 well plates. Their average fluorescence profiles were assessed, and a concentration dependent reduction of photoluminescence was observed between 5.0– 0.234 mM hexavalent chromium concentrations (Fig. 2). To quantitatively verify these findings, a Stern-Volmer plot comparing hexavalent chromium concentration with relative fluorescence output (as a fraction of the water control) was conducted. Results show that fluorescence quenching follows a linear trend in the presence of low Cr^6+^ concentrations between 0.234–1.875 mM (R^2^ value of 0.9979) (Fig. 3a and b). At higher concentrations (Cr^6+^ concentrations between 1.875–5.000 mM), the linear trend shifts indicating a move from monolayer Cr^6+^ adsorption to a multilayer adsorption mechanism [7]. The quantitative limit of Cr^6+^ detection within this assay is therefore 0.234 mM. Low variations in standard error reinforce the repeatability and accuracy of the measurements. This hydrogel-QCD-nanocomposite platform may be used with different QCD nanoparticles to select for different anolytes of interest to different detection limits.
